# Recall for postpartum follow-up of women with gestational diabetes mellitus: Climbing a mountain

**DOI:** 10.1186/s40200-016-0245-7

**Published:** 2016-07-02

**Authors:** Hajieh Shahbazian, Sedigheh Nouhjah, Shayesteh Jahanfar, Morteza Nasiri

**Affiliations:** 1 Health Research Institute, Diabetes Research Center, Ahvaz Jundishapur University of Medical Sciences, Ahvaz, Iran; 2 School of Population and Public Health, British Columbia University, Vancouver, Canada; 3 Department of Operating Room, Paramedical School, Qom University of Medical Sciences, Qom, Iran

Dear editor;

Early postpartum follow-up of women with a history of gestational diabetes mellitus (GDM) provides an opportunity to assess health condition of mothers as well as their offspring health [1]. GDM is considered as the strongest predictor of type 2 diabetes, thus follow-up of women with recent GDM can lead to early diagnosis and management of diabetes and other metabolic disorders outcomes [2].

In developed countries, a low postpartum return rate of women with GDM was reported for assessment of blood glucose [3]. In spite of high prevalence of GDM in Iran [4], evidences about postpartum return rate of women with this disorder and its predicators are not documented adequately.

Given the low postpartum return rate of women with GDM for oral glucose tolerance test and high use of mobile phone in Iran, we compared the effects of two methods of Short Message Service (SMS) text and telephone contact reminders on doing of oral glucose tolerance test (OGTT) for diagnosis of type 2 diabetes among women with GDM to design a novel method for recall of postpartum follow-up. We entered all of women with recent GDM who registered for screening of congenital hypothyroidism of their newborn early days after delivery in two main referral centers of Ahvaz, southwestern of Iran. Totally 476 women with abnormal glucose test in their recent pregnancy were evaluated for eligible criteria during January to March 2015. Among those, 156 women (residents of rural area, no available laboratory records of glucose status during pregnancy, cases of pregestational diabetes, early move out of Ahvaz after parturition, women with literacy problem, no access to mobile phone) were excluded and other 320 women that had inclusion criteria were assigned to receive either 4 SMS texts (*n*= 160) or 4 telephone contacts (*n*= 160) with the same dialog during 3–5 days to 6–12 weeks after delivery. SMS texts were sent to women, their husband or both. We asked all women in both groups to return for completion OGTT (a 75-g 2-h oral glucose tolerance test) during 6–12 weeks after delivery. Totally, 90 women (28.12 %) returned and performed OGTT during study period. Among those, 55 women (34.37 %) were in telephone contacts group and 35 women (21.87 %) were in SMS texts group. We concluded that postpartum return rate was higher in women with telephone contacts intervention.

Based on the results of this study, we continued recalling of women in life after gestational diabetes Ahvaz (LAGA) cohort study (IR.AJUMS.REC.1394.252) to compare potential metabolic outcomes in women with GDM (impaired glucose tolerance, diabetes type 2, lipids abnormality and metabolic syndrome) with healthy women. During 6 months recall from March 2015, we registered 1500 pregnant women (after 30 weeks’ gestation) who were referred for prenatal care to Ahvaz public health centers or Ahvaz private clinics to return for postpartum follow-up early after delivery and 2 years afterwards, in baseline visit. We used different methods of communication to recall women to participate. Invitation was sent via social networks (telegram and what’s up), mobile and direct contact via phone for women, their husband or the first relatives who their phone number was available. The baseline visit took place in 25 public and private health centers, and eligible women were invited to refer to cohort study center (Ahvaz Golestan hospital). Until now, data were collected in the first 6–12 weeks after delivery and data collection will continue until March 2017. Data collection tool in this study is a 180-item questionnaire including socio-demographic details, risk factors of gestational diabetes and its potential outcomes, medical and reproductive history, anthropometric measurements, physical activity, dietary intake, and breast feeding. Blood samples were collected in 6–12 weeks postpartum and will repeat annually within the first 2 years after delivery.

After excluded 200 mothers who could not meet study criteria, 1300 eligible women (800 women with GDM and 500 women with normal blood glucose) enrolled. Totally, 230 women (17.69 %) liked to participate in postpartum follow-up. Among those, 180 women (22.5 %) were with GDM and 50 women (10 %) were without GDM. Of women with GDM, 40 cases (5 %) didn’t not return to offered follow-up cohort study center, but reported uptake a test to check their glucose status which ordered by their physician in private clinics. Of those, 32 women (80 %) checked their glycemic status with fasting blood glucose (FBS) and the rest, uptake an OGTT during first weeks after delivery. Overall, return for follow-up and attendance for OGTT was low in early postpartum period in both our studies. It seems that perceived barriers for attendance and checking health status of women with GDM are stronger than the perceived risk of diabetes mellitus and other metabolic outcomes in the future [5].

Based on our experiences, it seems that poor communication between the public and private health sectors, inadequate referral system, lack of a national data registry and integrated health information system in addition to cultural and patient-related barriers are some challenges facing us for follow-up and lead to low rate of follow-up. These problems with controversies in screening, treatment and others concern make this process arduous. So, further research is suggested to explore the influencing factors for postpartum attendance with different methods. Also, it is recommended that with using experiences of other countries in this regard, novel strategies be taken by authorities.

## Abbreviations

FBS, Fasting glucose test; GDM, Gestational diabetes mellitus; LAGA, Life after gestational diabetes Ahvaz; OGTT, Oral glucose tolerance test (OGTT); SMS, Short message service
